# Developing an agenda for research about policies to improve access to healthy foods in rural communities: a concept mapping study

**DOI:** 10.1186/1471-2458-14-592

**Published:** 2014-06-12

**Authors:** Donna B Johnson, Emilee Quinn, Marilyn Sitaker, Alice Ammerman, Carmen Byker, Wesley Dean, Sheila Fleischhacker, Jane Kolodinsky, Courtney Pinard, Stephanie B Jilcott Pitts, Joseph Sharkey

**Affiliations:** 1 Nutritional Sciences, University of Washington, 98195, Box 353410, Seattle, WA, USA; 2 Center for Public Health Nutrition, University of Washington, 98195, Box 353410, Seattle, WA, USA; 3 Battelle, Health & Analytics, 1100 Dexter Avenue N., Suite 400, 98109 Seattle, WA, USA; 4 Department of Nutrition, Center for Health Promotion and Disease Prevention, University of North Carolina at Chapel Hill, CB# 7426, 1700 MLK/Airport Rd, Room 239, 27599–7426, Chapel Hill, NC, USA; 5 Department of Health and Human Development, Montana State University, 222 Romney Gym, 59717 Bozeman, MT, USA; 6 United States Department of Agriculture Food and Nutrition Service, Office of Policy Support, Food, Nutrition and Consumer Services Headquarters, 3101 Park Center Drive, 22302 Alexandria, VA, USA; 7 Division of Nutrition Research Coordination, National Institutes of Health, Two Democracy Plaza, Room 635 6707 Democracy Boulevard MSC 5461, 20892-5461 Bethesda, MD, USA; 8 Department of Community Development and Applied Economics, University of Vermont, 202 Morrill Hall, 05405 Burlington, VT, USA; 9 Gretchen Swanson Center for Nutrition, 8401 W Dodge Rd, Suite 100, 68114 Omaha, NE, USA; 10 Department of Public Health, East Carolina University, 600 Moye Blvd., Mailstop 660 Lakeside Annex Modular Unit 8, Room 126, 27834 Greenville, NC, USA; 11 Department of Health Promotion and Community Health Sciences, Texas A&M Health Science Center, School of Public Health, MS 1266, 77843-1266 College Station, TX, USA

**Keywords:** Rural populations, Nutrition, Food systems, Food access, Policy

## Abstract

**Background:**

Policies that improve access to healthy, affordable foods may improve population health and reduce health disparities. In the United States most food access policy research focuses on urban communities even though residents of rural communities face disproportionately higher risk for nutrition-related chronic diseases compared to residents of urban communities. The purpose of this study was to (1) identify the factors associated with access to healthy, affordable food in rural communities in the United States; and (2) prioritize a meaningful and feasible rural food policy research agenda.

**Methods:**

This study was conducted by the Rural Food Access Workgroup (RFAWG), a workgroup facilitated by the Nutrition and Obesity Policy Research and Evaluation Network. A national sample of academic and non-academic researchers, public health and cooperative extension practitioners, and other experts who focus on rural food access and economic development was invited to complete a concept mapping process that included brainstorming the factors that are associated with rural food access, sorting and organizing the factors into similar domains, and rating the importance of policies and research to address these factors. As a last step, RFAWG members convened to interpret the data and establish research recommendations.

**Results:**

Seventy-five participants in the brainstorming exercise represented the following sectors: non-extension research (n = 27), non-extension program administration (n = 18), “other” (n = 14), policy advocacy (n = 10), and cooperative extension service (n = 6). The brainstorming exercise generated 90 distinct statements about factors associated with rural food access in the United States; these were sorted into 5 clusters. *Go Zones* were established for the factors that were rated highly as both a priority policy target and a priority for research. The highest ranked policy and research priorities include strategies designed to build economic viability in rural communities, improve access to federal food and nutrition assistance programs, improve food retail systems, and increase the personal food production capacity of rural residents. Respondents also prioritized the development of valid and reliable research methodologies to measure variables associated with rural food access.

**Conclusions:**

This collaborative, trans-disciplinary, participatory process, created a map to guide and prioritize research about polices to improve healthy, affordable food access in rural communities.

## Background

Across the life course, access to nutrient-rich food that supports a healthy and active life is an important determinant of population health [1,2], but there are disparities in accessing healthy, affordable foods across income levels, among certain race/ethnic minority groups, and between rural and urban communities [3-7]. Although rural areas vary widely across the United States [8-10], rural residents generally consume fewer health-promoting foods like fruits and vegetables compared to urban or suburban residents [11]. Rural communities also face disproportionately higher risk for nutrition-related chronic diseases such as obesity when compared to urban residents [12-16]. Indeed, obesity prevalence is 39.6% among rural adults compared to 33.4% among urban adults, and remains significantly higher after controlling for demographics, diet, and physical activity [12]. Even among children, living in rural versus urban communities is associated with being overweight or obese [17].

While “food access” is complex and multi-faceted, evidence suggests individuals’ or communities’ ability to access healthy, affordable foods is influenced by affordability, availability, cultural acceptability, geographic proximity (e.g., how close a retail food outlet is to residential areas), and mobility and transportation options [3,4,18-21]. Research examining the associations between the food environment and health continues to grow [22,23]. To date, many studies find that residents of rural communities face disproportionate challenges to accessing healthy, affordable foods due to limited infrastructure, long distances to food outlets, and fewer healthy options [20,24-29]. Traditional grocery stores are becoming scarce in some rural communities [30], and those that remain may offer fewer healthy food options [31-33]. Some rural residents shop at local convenience stores (which typically offer more processed and less fresh food) and travel longer distances less frequently for groceries [18,33-35]. Despite these challenges, rural residents continue to feed their families through a complex arrangement of grocery stores and alternative or non-traditional food sources such as dollar stores, mass merchandisers (e.g., Walmart), convenience stores, fast food restaurants, mobile venders, and flea markets [36-38], as well as gardening, hunting, bartering, and reliance on neighbors and friends [18,21,38]. To compensate for transportation and proximity challenges, rural families may plan their food shopping around multi-purpose trips into regional hubs or urban centers (a practice known as “trip chaining”) [36], and store or freeze food they purchase in bulk [21].

National, tribal, state, and local efforts increasingly target improved access to healthy foods using a range of environmental and policy strategies such as retail food financing initiatives and land use regulations [39-46]. Most of these initiatives are focused on urban communities [47], and because rural food access appears to differ from urban food access, some proposed policy strategies may not address the needs of rural communities [48,49].

Given the interrelated and multi-level determinants of rural food access [11,45,50-52], a comprehensive and systematic approach is needed to plan research to support the development of effective rural food policies. To date, rural food research has not emphasized the impact of policy changes on rural food access, but has focused on individual-level topics such as factors influencing food choice [21], disparities [49], and trip chaining patterns [53,54], and community-level influences such as non-traditional food retailers [35,38,55], food venue types [28,56], and rural culture and context [47,57]. In concert with extant research, existing conceptual models of food access [2,58] do not fully address the influence of macro-level policies on the food choices of rural residents. Thus, there is a need for a detailed map to guide systematic examinations of the multiple levels of determinants of access to healthy foods in rural areas, and a need to prioritize potential research on the policies that might be applied to address these determinants.

This study aims to identify knowledge gaps and policy research needs that have the greatest potential for improving access to healthy, affordable foods in rural communities in the United States through a concept mapping process.

## Methods

### Nutrition and Obesity Policy Research and Evaluation Network Rural Food Access Workgroup

This work was conducted by the Rural Food Access Workgroup (RFAWG) of the Nutrition and Obesity Policy Research and Evaluation Network (NOPREN). NOPREN is funded by the United States Centers for Disease Control and Prevention (CDC) to conduct transdisciplinary nutrition- and obesity-related policy research and evaluation along a policy change continuum that includes policy identification, development, and evaluation [59]. In 2011, NOPREN members and collaborators formed a NOPREN Rural Food Access Working Group (RFAWG), which aims to share resources and conduct collaborative research that informs policy efforts to promote nutrition and healthy food access in rural settings ( http://www.hsph.harvard.edu/nopren/rural-food-access-working-group/). RFAWG members represent diverse geographic regions across the United States and a range of disciplines including public health, nutritional sciences, agricultural extension, rural sociology, food systems, economics, and public health law. Following a review of existing conceptual models of food access, the group identified the need for a systematic approach to plan rural food access policy research. The group sought an approach that would prioritize investigation of the most influential determinants of rural food access and the policies that could address these determinants.

Concept mapping is a multi-step participatory mixed methods approach used to solicit and collect, structure, and prioritize ideas [60]. *Concept Systems Global*, a proprietary online system for data collection and analysis, and *Concept Systems Core* software have been used by researchers and practitioners from higher education, public health, medicine and health care, nursing, tobacco control, cancer research, violence prevention, suicide prevention, youth development, community building, family support, scale development, psychology, and social welfare, to create concept maps to support strategic planning, needs assessment, evaluation, and research [61]. Because proximity on the resulting concept maps indicates how often respondents sort ideas into similar categories, the maps and figures that result from the process provide visual representations of the relationships between ideas and allow for group level interpretation and discussion about complex systems. Concept mapping has been used by NOPREN’s “sister” network, the Physical Activity Policy Research Network (PAPRN), to identify the highest priorities for research to support effective policy approaches to increase physical activity in the population [62].

A core group of eleven members of RFAWG collaborated on the concept mapping process. The methodology involves a step-by-step process: 1) identify participants, 2) develop a “prompt statement” to aid in soliciting ideas, 3) generate ideas through brainstorming, 4) structure the ideas through rating and sorting, 5) analyze the rating and sorting data, and 6) interpret the data. Using mean ratings, multidimensional scaling, and hierarchical cluster analysis, researchers created visual output in the form of maps and charts that were used to interpret the data. The University of Washington Institutional Review Board reviewed and approved all study protocols.

### Concept mapping process

#### Identify participants

Members of the RFAWG identified professionals with expertise in rural food access in the United States using a combination of nomination and snowball sampling. These experts included RFAWG members and those they referred, as well as professionals identified through authorship of practice-oriented and research literature and organizations with a reputation for working on issues relating to rural food access. By the end of the project, a total of 203 experts had been invited to participate in at least one of the data collection phases. The participants included a mix of researchers, government officials, and practitioners from various regions of the country. Potential participants received an email message that described the study purpose and the three activities (Brainstorming, Rating, and Sorting), and invited them to go to the study’s web site to participate in the concept mapping process. Upon initial log-in to the website, participants could respond to eight close-ended demographic questions.

### Data collection

#### Develop a prompt statement

Members of the RFAWG developed and tested the brainstorming prompt statement: “One thing that does or could make a difference for access to healthy food in rural areas is…” to generate brainstormed responses. The prompt statement was intended to solicit ideas related to barriers, facilitators, and existing or possible policies and programs related to rural food access.

#### Generate ideas through brainstorming

The brainstorming screen instructed participants to enter as many responses to the prompt statement as they desired, and listed all ideas suggested by other respondents to date. Instructions specified that participants did not need to type something that others had already suggested. The data collection period for brainstorming lasted from June 6, 2012 through June 29, 2012. Seventy-five participants (39% of the 192 invited) engaged in the brainstorming activity.

Three core group researchers (EQ, DJ, MS) used a process recommended by Kane and Trochim [60] to synthesize the brainstormed list of 245 statements into a shorter list of statements. To do so, a researcher (EQ) assigned key words to each statement, sorted the statements based on the terms, and then printed the statements on cards. Researchers (EQ, DJ, MS) then physically sorted, stapled, and wrote on the cards as they considered related ideas. The researchers removed duplicate ideas, combined specific statements describing the same or overlapping ideas, and edited statements for common syntax and wording. For example, the phrases “reliable, affordable transportation options,” “better transportation options for families,” “improved transportation to supermarkets and other healthy food outlets in nearby urban centers,” and “being able to easily get to store(s) that sell what people want for a price they can afford” were combined into: “Access to reliable, affordable and efficient transportation that links families to supermarkets and affordable food outlets”. This process resulted in a list of 90 synthesized statements. See Additional file 1.

#### Structure the ideas through rating and sorting

Experts were invited to rate each of the 90 statements on two dimensions – (1) policy priority and (2) research priority. As the experts viewed each statement online, first they were asked to, “Please read the following statements of things that do or could make a difference for access to healthy food in rural areas. Then rate each one in terms of the priority it should receive for the *development of policies* that would support, promote, alleviate or otherwise address rural healthy food access.” Then they were asked to “rate each one in terms of the priority it should receive for *conducting further research* to better understand its relationship to rural healthy food access.”

Participants could select a response of zero through four on a Likert scale, where zero referred to “Not a priority for policy development” or “Not a priority for further research” and four referred to “Highest priority for policy development” or “Highest priority for further research.” Seventy-one (37%) of the 192 experts invited completed the ratings. Because the sorting activity was predicted to be more time consuming than the other activities, a subsample of 52 experts were invited to sort. Stakeholders were selected for this activity if they participated in the RFAWG or worked directly in the area of rural food access research, policy, or practice (e.g., not rural or nutrition issues more generally). The subsample was designed to be balanced in terms of researchers and practitioners. Thirty of the 52 invited stakeholders (58%) completed the sorting activity. Using the online platform, participants could click and drag each individual statement from a list on the side of the screen into piles that they created. Instructions specified that participants should create “piles” based on perceived similarities and not create piles for “miscellaneous” statements. Both the sorting and the rating activities were available online for approximately one month.

#### Analyze the rating and sorting data

Visual maps of statement clusters were created with the *Concept Systems Inc. Core* software (version 4.0, Ithaca, NY) using similarity matrix calculations. The software calculated how many times participants sorted each pair of statements into the same cluster and used multidimensional scaling techniques to position statements in relation to one another based on how frequently they were paired during the cluster activity [61]. The proprietary software also provides “bridging value” indices for each statement to aid in interpretation. Lower bridging values indicate that the statement is “anchored” to or frequently sorted with those around it; higher values indicate that the statement is “bridging” or was paired by many respondents with statements further away.

Using hierarchical cluster analysis, the software partitioned the points on the point map clusters. Researchers determined the most intuitively accurate and useful number of clusters using the process recommended by Kane and Trochim [60]. Researchers initially considered maps containing as few as 4 and as many as 20 clusters. During the interpretation session described below both a more general 5 cluster map and a more detailed 13 cluster map were examined. The 5 cluster map was considered to be visually superior in summarizing the results of the concept mapping process. Concept mapping experts recommended considering “the judgment and sense of the participants to refine and revise the cluster analysis results (pg. 104) [60],” so the research team made modifications to the final map based on a combined assessment of statements’ bridging values and their qualitative relation to one another.

Finally, the researchers created *Go Zone* matrices and *Pattern Match* charts*. Go Zone* matrices assign x- and y-axes to sets of rating data where each statement is assigned coordinates based on its respective mean rating. Overlaying lines corresponding to the mean rating for each axis divide the graph into four quadrants (low/low, low/high, high/low, and high/high). Under the assumption that high ratings are ideal, the high/high quadrant is considered the *Go Zone* as it contains those ideas rated most highly on both criteria (of development of policies and further research). Pattern match charts compare each cluster’s mean rating criteria across participant expert professional roles. The research team used ANOVA to test for differences across the three categories of researchers, practitioners, and cooperative extension professionals.

#### Interpret the data

In September 2012, the 11 authors convened for an in-person meeting to review and interpret data output, and to identify priorities for future NOPREN research. Following the presentation of results, the authors split into three small groups. Each group focused on a *Go Zone* chart for one or two of the five “domains” with the goal of identifying priority areas for policy research in each domain. During the discussion, these experts considered the feasibility of conducting policy research in each area, the potential impact on disparities and other important outcomes, and the degree to which potential research topics were within the scope of rural food researchers.

Results of the interpretation meeting were captured through detailed descriptions of the proceedings and structured notes from each small group. Key themes and recommended policy research priorities were synthesized from these documents.

## Results

### Study participants

As presented in Table 1, participants who responded to the survey represented diverse regions of the United States, and diverse stakeholders in the broad field of rural food access policy and research. In several cases, participants did not use the username and password they had created in the first activity to log-back for the later activities, instead creating a second account. In these instances, they may have responded differently to the demographic questions, so demographic summaries are most accurately presented by each activity rather than for the project as a whole.

**Table 1:** Characteristics of the respondents

	**Brainstorming phase**	**Rating phase**	**Sorting phase**
	**n = 75**	**n = 71**	**n = 30**
**Region**			
Northwest/West	26	24	10
Midwest	7	6	2
South/Southeast	18	21	10
Northeast	11	7	3
Nationwide	11	13	5
No Response	2	0	0
**Field of Expertise**			
Agriculture/food production	4	5	2
Public health/nutrition	29	35	14
Food retail	1	0	0
Community/economic development	5	2	1
Food security/hunger	11	12	6
Schools/child care	1	3	0
Food system development	14	7	4
Other	8	7	3
No response	2	0	0
**Role**			
Cooperative Extension Staff/Researcher	6	10	1
Non-extension researcher	27	29	13
Non-extension program director/staff	18	14	6
Policy advocate	10	9	3
Other	12	9	7
No response	2	0	0

### Concept map

**Figure 1:**
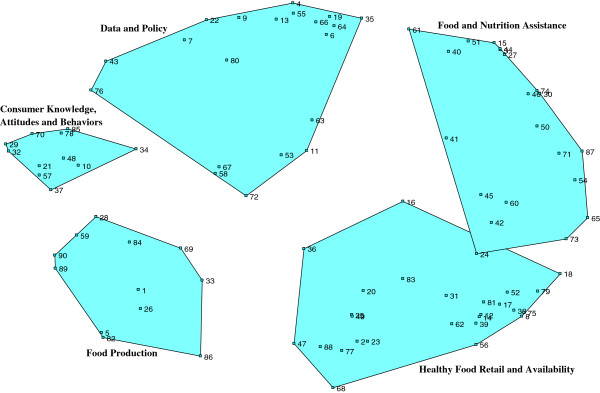
Concept map.

The map includes five large clusters that each include between 11 and 26 statements: (1) *Food and Nutrition Assistance*; (2) *Healthy Food Retail and Availability*; (3) *Food Production*; (4) *Consumer Knowledge, Attitudes and Behaviors*; and (5) *Data and Policy*. See Figure 1. Researchers decided to move one statement, “Purchasing groups that link child care programs, schools and long-term care facilities to affordable foods” from the *Healthy Food Retail and Availability* cluster to *Food and Nutrition Assistance* cluster based on the qualitative fit of the statement with others in the two domains.

### Pattern match by respondent professional roles

Overall, there were significant differences in the policy priority ratings by respondent role (p = .005). While the mean rating of the statements’ priority for policy development was 2.45 for program directors and advocates and 2.45 for extension researchers and staff, the mean rating of policy priority by non-extension researchers was only 2.19. Although it was not statistically significant, the trend for prioritizing policy research was the opposite, with non-extension researchers rating the need for further policy research higher than the other respondents.

### Go zones

Table 2 provides the statements plotted in the *Go Zones* for each of the five clusters (i.e., rated above the mean for both policy development and policy research). Of all 90 statements, the one rated highest for both research and policy was about the need to develop food access solutions that are particular to rural areas. Policies that could help to support stronger local farm and food system economies such as addressing food safety regulations that impact the ability of farmers to sell their products locally, rural economic development, “combining forces” for increased purchasing, distribution, and selling power were also highly ranked in the *Go Zone.* In addition, several statements that addressed food retail in rural areas were ranked as priorities for both policies and research.

**Table 2:** “Go Zone” statements prioritized highest for research and policy development within each of the five domains based on respondent ratings

**Domain**	**Statement #**	**Statements**	**Research rating**	**Policy rating**
Data and policy	6	Food safety policies that are feasible for small farmers and business to implement, including those related to school meals and school gardens	3.20	3.23
	72	Economic development that supports the role of local food in community economic vitality	3.02	3.15
	80	Economic development that supports living wage jobs that improve the purchasing power of low-income rural residents	2.85	3.32
	55	Policymakers understanding the barriers to obtain healthy food in rural communities	2.82	3.46
	35	USDA procurement regulations prioritizing healthy food purchases	2.69	3.33
	64	Sustained coalition-building on Farm Bill policy between community food security, anti-hunger, rural development, public health, dietitians, etc.	2.61	3.31
	63	Farmers that wish to grow produce have equitable access to subsidies, crop insurance, agricultural loans, and technical assistance	2.70	3.42
Food and nutrition assistance	42	Developing food access solutions that are particular to rural areas	3.38	3.40
	74	Accessibility of food assistance programs in rural areas	2.77	3.10
	71	Schools having access to fresh fruit and vegetable snacks	2.64	3.42
	24	Purchasing groups that link child care programs, schools and long-term care facilities to affordable foods	2.69	2.90
	60	Schools purchasing produce directly from farmers	2.61	2.99
	46	SNAP/EBT (food stamps), WIC and Seniors Farmers Market coupons are accepted at all forms of rural food retailers	2.52	3.29
	3	Benefit levels of WIC fruit and vegetable vouchers	2.50	3.00
	87	School nutrition programs offering culturally appropriate food choices	2.48	2.90
	61	Policymakers understanding the economic benefits of streamlining the SNAP benefits process and getting more SNAP benefits into rural areas	2.48	2.93
Consumer knowledge, attitudes and behaviors	57	Young people having experiences with healthy eating, gardening and activity choices in schools and communities	2.52	2.84
	70	People understanding and believing that healthy food results in more than just health outcomes, including improved grades, and stronger businesses and workforces	2.63	2.59
	10	People knowing how to prepare low cost, healthy, farm-fresh foods safely	2.43	2.59
	78	Students having opportunities to learn about agriculture, science, technology, engineering, math, and food production	2.16	2.44
	37	Access to information and guidance about growing, preparing and securing healthy and affordable foods	2.15	2.54
Healthy food retail and availability	20	Local and regional food systems that have the capacity to combine forces for increased purchasing, distribution and selling power	3.24	3.14
	31	Systems and options that bring foods directly to rural consumers	3.08	3.09
	8	Corner stores and small retail stores that sell sufficient and diverse healthy food options	3.00	3.06
	52	The location of markets, produce trucks, farm stands and food carts in accessible locations in town or “rural hubs”	3.00	2.93
	39	Diversity of food retail options in rural areas	2.92	3.01
	16	Cities and towns that support farmers markets and local foods by reducing logistical barriers to their promotion	2.89	3.13
	83	Infrastructure that allows for safe and economically feasible transport of goods to rural markets	2.77	2.93
	56	Access to reliable, affordable and efficient transportation that links families to supermarkets and affordable food outlets	2.75	3.07
	17	Rural areas with a sufficient number of affordable small markets and grocery stores	2.72	3.25
	38	Retail distributors carrying and delivering a variety of healthy food choices to rural areas	2.66	2.94
Food production	82	Fruit and vegetable farm workers receiving fair wages and working conditions	2.36	3.15
	28	Solutions that identify and build from rural community/family strengths	2.77	2.78

**Figure 2:**
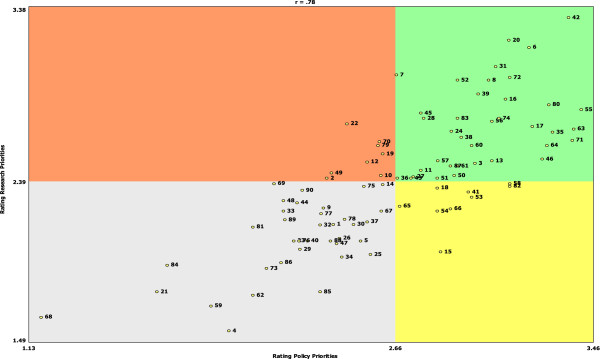
“Go Zone” chart.

Figure 2 shows the *Go-Zone* chart for all of the statements. It is noteworthy that, as shown visually by the chart, most statements had similar rankings for both policy priority and research priority. There were some exceptions to this; for example, the idea of a streamlined application processes and eligibility rules for the Supplemental Nutrition Assistance Program (SNAP) (statement 15) was rated higher for policy priority than for a research priority, and understanding the determinants of food insecurity (statement 6) was rated higher for research than for policy. The statements that were ranked among the lowest for both policy and research addressed more individual characteristics of rural residents such as their travel patterns (statement 68) or social standing (statement 21) and social-political factors in rural areas such as traditional land use patterns (statement 84), out-migration from rural areas (statement 59), and access to political decision-making (statement 4).

### Interpretation session: key themes and research priorities

During the interpretation session policy research priority areas were determined to be economic development and viability and consumer purchasing power food; food production capacity; retail availability and shopping patterns; nutrition assistance program adaptations; cooperative extension impact on access; role of emergency food services; and rural research methods development. Table 3 provides details. Priorities determinations were based on the “*Go Zone*,” categories in conjunction with the expert opinion of the core research group about the potential feasibility, scope, and impact of research in these areas.

**Table 3:** Rural food access policy research priorities based on NOPREN RFAWG* core group interpretation

**Research Priority**	**Domain**	**Description**
Economic development, viability and consumer purchasing power in rural communities	Data and Policy	How can economic development efforts, particularly through food producers and entrepreneurs, influence consumer purchasing power and behaviors? How do various food safety regulations impact market concerns? What policies help or hinder rural economic development?
Food and nutrition assistance program adaptations for rural communities	Food and Nutrition Assistance	What can we understand about barriers and facilitators for food assistance programs and other nutrition support services in rural areas? This information would inform needed policy adaptations and involve conducting translational activities.
Role of emergency food services in rural communities	Food and Nutrition Assistance	What can we understand about how rural communities address emergency food needs in rural communities? To what extent is service delivery in rural areas cost-effective? What are the best practices and how should these be disseminated?
Cooperative extension impact on access	Consumer Knowledge, Attitudes and Behaviors	Evaluation of some educational programs in rural communities, such as SNAP-Ed, have been limited. How can researchers partner with outreach initiatives to learn how these efforts support policy objectives? For example, what is the role of cooperative extension in large policy and environmental change initiatives such as Communities Putting Prevention to Work?
Rural retail availability and shopping patterns	Healthy Food Retail and Availability	Rural communities have various retail options (e.g., mass retailers, bulk stores, dollar stores, corner stores, grocery stores, underground food economies). How do these options influence consumer shopping habits? What policies impact rural retail options?
Rural food production capacity	Food Production	Rural communities used to grow their own food and now no longer do. What capacity (e.g., social, financial, technical) do rural communities have and lack for food production? What policies and practices can improve food production capacity for rural residents?
Rural research tool and method development	Cross-cutting (relevant to all domains)	There is a need for projects that inform the development and adaptation of best practice and research measurement tools appropriate for rural demographics and geography. Food access studies conducted in urban and suburban settings can also be replicated in rural contexts.

*Nutrition Obesity Policy Research and Evaluation Rural Food Access Workgroup.

During the interpretation session it became clear that some statements that emerged in response to the initial prompt reflect important conceptual and contextual ideas pertaining to food access issues that may inform study approaches, but may not be appropriate for policy research specific to food access. Therefore, these statements were removed from the groups in which they were initially placed on the cluster map. These statements related to the importance of identifying and building from existing rural strengths (statements 28 and 42), limitations imposed by a group’s social standing (statement 21), and the importance of out-migration among rural populations (statement 59). *Consumer Knowledge, Attitudes and Behavior* statements were least relevant to potential policy solutions that focus on food environments, but the group acknowledged that there is a need for research about ways in which publicly funded programs and services support changes in rural residents’ food and nutrition knowledge, attitudes and behavior, and these statements remained in the map.

## Discussion

Food access plays a significant role in the nutritional health of the population, and policy strategies are increasingly recognized as important to assuring this access. Such policies must be contextually appropriate [47,57]. Many of the clusters appearing in the concept maps are similar to extant conceptual models that describe food choice, food security, and nutritional environments [2,63], but within each cluster there are additional ideas that are unique to rural areas. These can provide direction to rural food policy researchers and are currently serving as a basis for the ongoing work of the NOPREN RFAWG.

Many statements within the clusters illustrate ways the concepts may have a different context in rural areas. For example, transportation concerns are referenced frequently in relation to several clusters. Transportation is often needed for travel to food sales outlets (*Healthy Food Retail Availability*) and to participate in some state and federal food and nutrition assistance programs *(Food and Nutrition Assistance)*; transportation is also needed to distribute healthy items to rural stores (*Local Food Systems)* and to move agricultural products to market (*Price and Community Economic Development*). Collectively, the clusters illustrate the complexity of the inter-related and multi-level factors associated with rural food access. For example, policies that incentivize healthy food retail may not be effective if they simply lead to the opening of new stores because food access in rural communities is associated with other issues: food distribution systems, pricing of healthy foods, and food purchasing behaviors. Moreover, opening new stores in rural communities might have unintended consequences such as increased access to processed food and economic vulnerability for existing local storeowners [4].

Results from this study are informing practice and research about the highest priority work that needs to be done to improve availability of healthy food in rural areas through policy and environmental change [64]. For example, several concepts related to rural economic development were rated as the highest priorities for both policy and policy research. Given the dual importance of food production to rural areas and the need for financial resources to access food in rural areas, economic development as a way of improving access to healthy foods in rural areas is a unique focus for policy research. A sub-group of RFAWG members have focused on this arena. The group conducted a literature review to examine four entrepreneurial food systems innovations (farmers’ markets, community supported agriculture (CSAs), farm to institution programs and food hubs) to determine whether innovations for aggregation, processing, distribution and marketing in local food systems: 1) enabled producers to make a living; 2) improved local economies; 3) provided local residents with greater access to affordable, healthy food; and 4) contributed to greater consumption of healthy food among residents [65].

An additional set of highly ranked statements are focused on healthy food retail and availability. The concept map *Go Zone* rankings indicated that the highest priority research could focus on small retail stores, innovative markets, trucks/carts and farm stands, and rural farmers markets. A sub-group of RFWAG researchers is starting to develop a research agenda in rural food retail. Initially, the group plans to focus on two areas – customers’ perceptions of rural retail and systems and policy influences on the movement of foods through rural communities.

The opportunity to come together in the RFAWG for this project and the ongoing research network has also focused researchers on the need to build rigorous methods for rural food policy research. To that end, a third RFAWG sub-group is expanding on previous findings [66] to adapt the Common Community Measures for Obesity Prevention” (COCOMO), a set of 24 recommended community-level obesity-prevention strategies for use in rural areas.

It is clear that by starting to consider research questions about economic viability of food retail and the inter-relatedness of community economic development and food access, researchers need to work across disciplines and secure meaningful community involvement [64,65]. Ideally, these findings serve as a call for multi-disciplinary and collaborative studies that look at the factors associated with rural food access across diverse populations. RFAWG plans to continue to use the concept map as a guide to developing appropriate, testable research questions about policy development and evaluation for food access in rural areas.

As with any study, there are important considerations worth noting when interpreting these findings. First, results reflect the group doing the study. This study describes rural food access and research priorities from the perspective of professionals, which may differ from those of rural residents. To address this limitation, RFAWG members have also been conducting focus groups of rural residents (personal communications, Carmen Byker, January 28, 2014; Wesley Dean, February 1, 2014; Stephanie Jilcott Pitts, January 21, 2014). In addition, many of the professional experts come from the field of public health. Perceptions from a different sample may result in different ideas about rural food access issues and their relative priority for policy development and future research. Reaching across food system “spheres” in this manner is a challenge for both food system and policy research. Finally, the study does not use a particular definition of “rural” or directly address the diversity of communities generally referred to as rural. The strengths of the study include the participatory design, mixed method data collection approaches, visual mapping products, and a sampling approach that aimed to capture perspectives of experts across diverse disciplines, geography, and researcher/practitioner points of view.

## Conclusion

This collaborative concept mapping project created a detailed map of clusters of 90 different statements that describe a diverse set of factors associated with food access in rural communities in the United States. Findings from this study have important implications for rural food policy researchers and practitioners because they define the context and determinants of rural food access and the policies that could be effective for rural communities. The maps and other findings can be used to establish a comprehensive research plan to build evidence to guide policy development.

## Abbreviations

NOPREN: Nutrition and Obesity Policy Research and Evaluation Network; RFAWG: Rural Food Access Workgroup; CDC: Centers for Disease Control and Prevention; SNAP-Ed: A nutrition education program for SNAP participants, United States Department of Agriculture Supplemental Nutrition Assistance Program; WIC: United States Department of Agriculture Special Supplemental Nutrition Program for Women, Infants, and Children.

## Competing interests

The authors have no competing interests to declare.

## Authors’ contributions

DJ conceived of the study and provided general oversight. EQ coordinated study activities. EQ, DJ, and MS conducted data analysis. All authors contributed to the study’s design, sample selection, and data interpretation. All authors contributed to, read, and approved of the final manuscript.

## Authors’ information

All authors participate in the Nutrition and Obesity Policy Research and Evaluation Network Rural Food Access Working Group (RFAWG). DJ and JS co-chair the group.

## Pre-publication history

The pre-publication history for this paper can be accessed here:

http://www.biomedcentral.com/1471-2458/14/592/prepub

## Supplementary Material

Additional file 1 **Concept Mapping Statements.** This file lists the 90 statements that participants rated and sorted into clusters. Statement numbers correspond to numbers presented in Table 2, Figure 1, and Figure 2. Click here for file
